# NKT Cells in Mice Originate from Cytoplasmic CD3-Positive, CD4^−^CD8^−^ Double-Negative Thymocytes that Express CD44 and IL-7Rα

**DOI:** 10.1038/s41598-018-37811-0

**Published:** 2019-02-12

**Authors:** Zhansheng Hu, Wen Gu, Yang Wei, Gang Liu, Shengli Wu, Tie Liu

**Affiliations:** 1 Immunology and Tumor Research Instituted, the First Affiliated Hospital, Xi’an Jiaotong University Health Science Center, Xi’an, Shanxi 710061 PR China; 2 The First Affiliated Hospital, Jinzhou Medical University, Liaoning, 121004 PR China; 3 Core Research Laboratory, the Second Affiliated Hospital, Xi’an Jiaotong University Health Science Center, Xi’an, Shanxi 710049 China; 4 Clinical Research Center, Guangdong Medical Collage, Zhanjiang, Guangdong 524001 China; 5 Department of Hepatobiliary Surgery, the First Affiliated Hospital, Xi’an Jiaotong University Health Science Center, Xi’an, Shanxi 710061 PR China

## Abstract

Although natural killer T cells (NKT cells) are thought to be generated from CD4^+^CD8^+^ (DP) thymocytes, the developmental origin of CD4^−^CD8^−^ (DN) NKT cells has remained unclear. In this study, we found the level of NK1.1 expression was highest in DN cells, followed by CD4 and CD8 (SP) and DP cells. The level of NK1.1 expression was highest in CD44^+^CD25^−^ (DN_1_) cells, after that CD44^+^CD25^+^ (DN_2_), finally, CD44^−^CD25^−^ (DN_3_) and CD44^−^ CD25^+^ (DN_4_) cells. Unexpectedly, cytoplasmic CD3 was not only expressed in SP and DP thymocytes but also in most DN thymocytes at various stages. The mean fluorescence of cytoplasmic and surface CD3 in DN cells was significantly lower than in mature (SP) T and NKT cells in the thymus and spleen. Interestingly, there were more NKT cells in DN-cytoplasmic CD3 expression cells was higher than in DN-surface CD3 expression cells. There were more CD3-NKT cells in DN_1_ thymocytes than in TCR-β-NKT cells. NKT cells expressed higher levels of IL-7Rα which was correlated with CD44 expression in the thymus. Our data suggest that T cells and NKT cells follow similar patterns of expression with respect to cytoplasmic and surface CD3. Cytoplasmic CD3 could be used as a marker for early stage T cells. Both cytoplasmic CD3 and surface CD3 were expressed in mature T cells and immature T cells, including the immature cytoplasmic CD3^+^ surface CD3^−^ and surface CD3^+^TCR-β^**−**^ cells in DN_1_-NKT thymocytes. CD44 could be used as an additional marker of NKT cells which may originate from cytoplasmic CD3-positive DN thymocytes that express CD44 and IL-7Rα in mice.

## Introduction

T lymphocytes expressing NK cell lineage markers (NK1.1, CD56) are referred to as NKT lymphocytes and have characteristics of both T and NK cells^1^. NKT cells are a unique and small subset of regulatory T cells. NKT cells recognize glycolipid antigens, such as α-galactosylceramide (αGalCer), bridge innate and adaptive immunity and modulate immune responses in autoimmunity, malignancies and infection^2–4^. NKT cells can produce large amounts of both Th1 and Th2 cytokines as an immediate response to TCR ligation^5,6^. However, NKT cells have also been shown to display cytotoxic activity, in a mechanism similar to that of NK cells^7^. In adult mice, subsets of immature double-negative thymocytes, termed DN_1_ and DN_2_, have NK-cell potential^8,9^. Previous studies demonstrated that T and NK cells were derived from a common precursor. Although NK1.1^+^ T cells may have a developmental pathway similar to that of T and NK cells, it has not been clear where NK1.1^+^ T cells branch off from this common pathway^10,11^. A previous study showed that NKT cells likely develop from DP cells^12^. Another precursor candidate of NK1.1^+^ T cells may be NK1.1 TCR cell population. Sato *et al*. demonstrated that the NK1.1 surface CD3ε population could differentiate into mature NK1.1^+^ T cells in the presence of IL-15^13^.

CD3 chains play essential roles in intracellular assembly, transport of TCR–CD3 complex to cell surface, receptor internalization and differential signal transduction. The pre-TCR and TCR contain four distinct signal-transducing subunits—CD3γ, CD3δ, CD3ε, and ζ_α^14–16^. CD3 chain specific transcripts are expressed as early as DN_1_ stage and almost all the DN_3_ thymocytes express high amounts of clonotype-independent CD3 complexes (CIC) in pro-thymocytes before the expression of pTα and TCRβ^17^. The intracytoplasmic (IC) domain of CD3 is essential for early thymocyte maturation and deletion of the CD3ε intracellular chains, but not the CD3γ or CD3δ intracellular domains results in a block in early thymocyte development^18^. While some previous studies show that intracellular CD3 are expressed as early as DN_1_ stage, and all CD25^+^ cells express intracellular CD3^19^, many other reports say that intracellular CD3 was first detected in CD25CD44^lo^ CD117^lo^ thymocytes^20^. The TCR lacks intracellular signaling domains, but interacts with CD3 subunits, which possess intracellular immunoreceptor tyrosine-based activation motifs (ITAMs) for phosphorylation^21,22^.

Interleukin (IL)-7 is a cytokine essential for lymphocyte development and survival, and stimulates the proliferation of thymocytes, and mature T cells through an interaction with its high affinity receptor (IL-7R) belonging to the hematopoietin receptor superfamily^23^. IL-7R consists of a common cytokine receptor γ-chain (γ_c_) and a unique IL-7R α-chain (IL-7Rα). It is reported that γ_c_-deficient mice lack thymic NKT cells^24^. In contrast, the proportion of NKT cells is not reduced in the IL-7- or IL-7Rα-deficient thymus, although absolute numbers of thymic NKT cells are drastically reduced. Matsuda *et al*. observed that IL-7/IL-15 doubly deficient mice exhibited more severe reduction in NKT cells than IL-15-deficient mice^25^. Nonetheless, the roles of the IL-7R in development of NKT cells in the thymus remain unclear, and little is known about the roles of IL-7Rα in different stages of T-cell development.

CD44 is a widely distributed cell surface marker and cell adhesion molecule in normal adult and fetal tissues. CD44 has the potential to participate in several processes associated with the induction of cell motility, activation of cell survival responses, and promotion of cell adhesion^26^. Various reports have implicated CD44 in the development and function of the immune system and its expression during a particular stage or in a subset of thymocyte progenitors suggests its functional involvement in the homing and thymic colonization of precursor cells^27^. CD44 and lymphocyte activation and its relatively high level of cell surface expression on NKT cells have been observed^28^. Our previous study demonstrated that CD44 expression positively correlated with Foxp3 expression and suppressive function of CD4^+^ T_regs_^29^.

In this study, we examined NK1.1, CD44, surface CD3, and cytoplasmic CD3 expression in thymocytes, and found that NK1.1 expression was correlated with CD44 and IL-7Rα expression in NKT cells. T and NKT cells developed from CD4^−^CD8^−^ T progenitor cells through different pathway.

## Materials and Methods

### Experimental animals

C57BL/6 mice were supplied by the Laboratory Animal Center of Xi’an’s Jiaotong University. Female mice were studied at 5 weeks old, and maintained under specific pathogen-free conditions. All studies were approved under the Institutional Animal Care and Use Committee at Xi’an Jiaotong University protocol (approval number: xjtu2018-005), and conformed to the Guide for the Care and Use of Laboratory Animals published by the US National Institutes of Health.

### Antibodies and flow cytometry

The antibodies were used for cell labeling in flow cytometric analysis (FACSAria III, BD Biosciences). Cells were derived from the murine thymus and spleen and blocked with mouse CD16/CD32 mAb. Then the cells were stained with PerCP-anti-CD8 (clone: 53-6.7), FITC-anti-CD4 (clone: RM4-5), APC-CY7- anti-CD44 (clone: IM7), PE-CY7- anti-CD25 (clone: PC61), FITC anti-CD11b (clone: M1/70)or PE-anti- TCR-β(clone: H59-597, Isotype: Armenian Hamster lgG2), PE-Anti- CD3 (clone:145- 2C11, Isotype: Armenian Hamser IgG1,κ), PE-anti- IL-7R(clone: A7R34, Isotype: Ret IgG2b,κ), and/or APC-anti-NK1.1 (clone:PK136, Isotype: Mouse (C3H × BALB/C) lgG2a,κ). For CD3 intracellular staining, the cells were fixed/permeabilized and then stained with PE-Texas Red anti-CD3 (clone: 145-2C11, Isotype: Armenian Hamster IgG1, κ). The antibodies were purchased from BD Biosciences and eBioscience.

### Detection DN cells by improved flow cytometry

TCR regulate T-cell proliferation and differentiation in the thymus and the periphery^30,31^. While flow cytometry is one of the most important research methods for examining T cell development, traditional flow cytometry cannot accurately predict percentages for cells that are expressed below a certain percentage or a certain amount. CD4^−^CD8^−^ DN cells comprise only 3–5% of the total thymocytes within the thymus^32^. We here used an advanced flow cytometry method to explore DN-T and T cell development in the thymus^33^. Furthermore, the expression of many genes, such as the *TCR-β* gene is very low in DN thymocytes; therefore, accurate detection of protein molecules in various stages of DN thymocytes by flow cytometry is challenging. As shown in Fig. S1. Therefore, using this improved the flow cytometry detection method (5 × 10^6^ thymocytes were collected for each sample). Moreover, lower expression protein molecules in each subpopulation of DN cells could be detected to reveal previously uncharacterized data on subsets of DN cells.

### Flow cytometric method for elimination of contaminating cells within DN thymocytes

Traditionally, contaminated cells (non–T-cell lineages) must be removed by specific blocking antibodies before detection of DN cells. We found cytoplasmic CD3 was expressed in the majority of DN thymocytes, and removed contaminating cells by the cytoplasmic CD3 gated (a detection software technology of flow cytometry) and then analyzed protein molecules in DN thymocytes (Fig. S2). The methods can be used to detect the DN thymocytes and remove contaminating cells (such as CD11b, B220).

### Statistical analysis

Results are presented as the mean and standard deviation. The software of GraphPad Prism was used in all analysis. More than three independent experiments were performed. The Tukey ‘ test was used to compare 3 or more means and a two-tailed unpaired *t* test was used to compare 2 groups. *P* ≤ 0.05 was considered to indicate a statistically significant difference between values. Statistically significant values are given in all figures.

## Results

### Surface CD3 and NK1.1 expression in thymocytes is higher within DN than DP thymocytes

**Figure 1:**
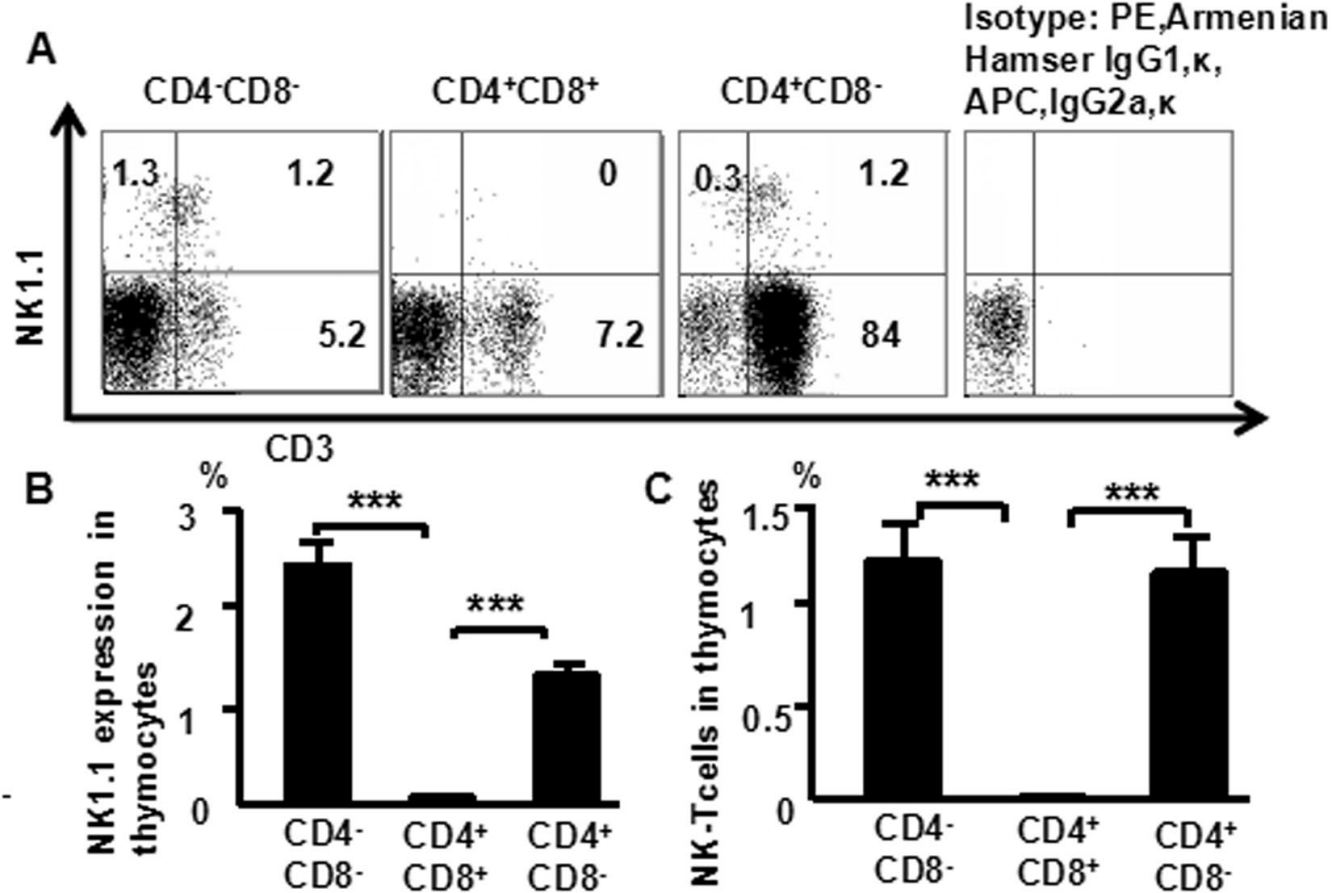
Surface CD3 and NK1.1 expression in thymocytes is higher within DN than DP thymocytes. Cells were stained with CD4 (FITC), CD8 (PerCP), CD44 (APC-CY7), CD25 (PE-CY7), NK1.1 (APC) and CD3 (PE) and then analyzed by flow cytometry. (**A**) CD3 and NK1.1 expression in DN, DP, and SP cells were analyzed; Data were pooled from three independent experiments and shown in the plot. (**B**) NK1.1 expression in DN, DP, and SP cells; (**C**) NKT cells in DN, DP, and SP cells. The data shows the percentage of total and live thymocytes in each cell subset, and are presented as the mean ± SD from three independent experiments. (***P < 0.001).

Cells from the murine thymus were stained with following antibodies in multiparameter flow cytometric analysis. CD8 (PerCP), CD4 (FITC), CD44 (APC-Cy7), CD25 (PE-Cy7), NK1.1 (APC), and CD3 (PE). NK1.1 expression is shown in (Fig. 1A). NK1.1 expression was higher in DN cells (2.5%) than SP cells (1.5%) and DP cells (0%), and there were more NKT cells in DN cells (1.2%) and SP cells (1.2%) than in DP cells (0%) (Fig. 1B,C). These data suggest that NKT cells develop from CD4^−^CD8^−^ T progenitor cells without the involvement of the CD4^+^CD8^+^ stage in the thymus.

### Surface CD3 and NK 1.1 expression is highest within DN_1_ and DN_2_ populations

**Figure 2:**
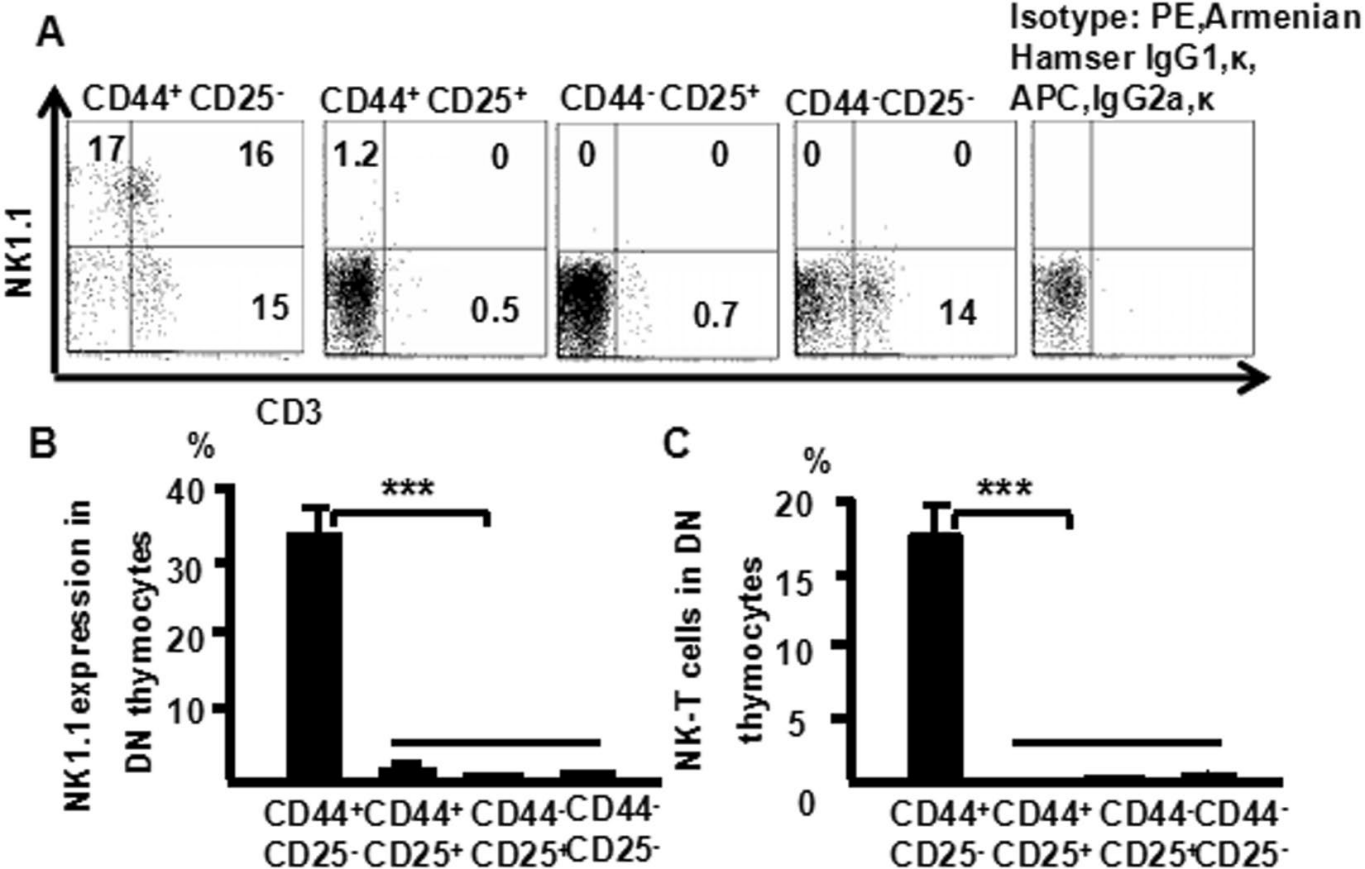
Surface CD3 and NK 1.1 expression is the highest within DN_1_ and DN_2_ populations. Cells were stained with CD4 (FITC), CD8 (PerCP), CD44 (APC-CY7), CD25 (PE-Cy7), NK1.1 (APC) and CD3 (PE) and then analyzed by flow cytometry. (**A**) CD3 and NK1.1 expression in CD44^−^CD25^−^, CD44^−^CD25^+^, CD44^+^CD25^−^, and CD44^+^CD25^+^ DN cells was analyzed. Data were pooled from three independent experiments and shown in the plot; (**B**) NK1.1 expression in DN cells; (**C**) NK1.1 expression in CD3^+^ DN cells. The data shows the percentage of total and live thymocytes in each cell subset, and are presented as the mean ± SD from three independent experiments. (***P < 0.001,**P < 0.01).

Natural killer T (NKT) cells are a unique subset of T cells that express both T cell receptor (TCR) and NKR-P1B/C (NK1.1; CD161) on their surfaces but at lower density than conventional T cells and NK cells, respectively^1^. The αβ-TCR is part of a multi-chain complex that is composed of the peptide–MHC-binding TCRα and β chains that are non-covalently associated with CD3 and TCR, which together are referred to as the CD3 complex^9^. To examine NKT cells in DN thymocytes, cells from the murine thymus were stained with CD8 (PerCP), CD25 (PE-Cy7), CD4 (FITC), CD44 (PE-Cy7), NK1.1 (APC), and CD3 (PE), and analyzed them by multiparameter flow cytometry (Fig. 2A). For DN cells from thymocytes, the highest expression level of NK1.1 was observed in DN_1_ cells (33%), followed by DN_2_ cells (1.2%), DN_3_ and DN_4_ cells (0%) (Fig. 2B). The number of NKT cells was much higher in DN_1_ (16%) than DN_2_, DN_3_, and DN_4_ cells (0%) (Fig. 2C). These data suggest NKT cell development may initiate from DN_1_ and DN_2_ without passing through DN_3_ or DN_4_ stages.

### Cytoplasmic CD3 expression is highest within DP and DN3 subpopulations of thymocytes

**Figure 3:**
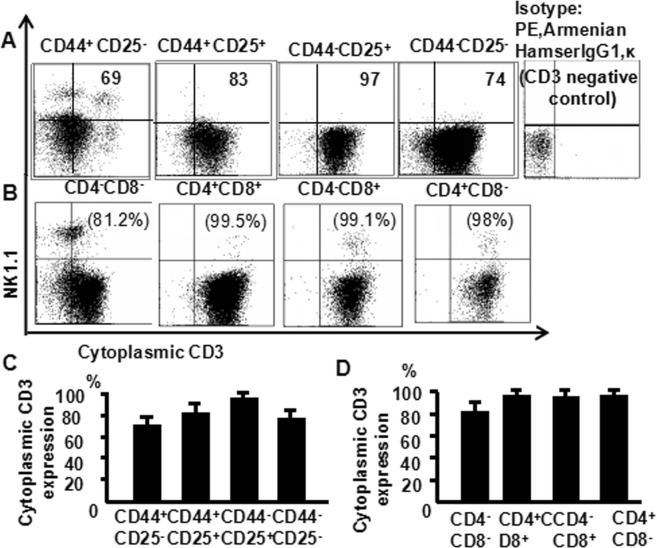
Cytoplasmic CD3 expression is the highest within DP and DN3 subpopulations of thymocytes. Thymocytes from naive mice were stained with CD4 (FITC), CD8 (PerCP), CD25 (PE-Cy7), CD44 (APC-Cy7), NK1.1 (APC) and CD3 (PE), and analyzed by flow cytometry. (**A**) Cytoplasmic CD3 expression in the different population of DN thymocytes; (**B**) Cytoplasmic CD3 expression in the different population of thymocytes; The pooled data from three independent experiments (**C**,**D**). The data shows the percentage of total and live thymocytes in each cell subset, and are presented as the mean ± SD from three independent experiments. (*P < 0.01).

Cells from the murine thymus were stained with CD8 (PerCP), CD25 (PE-Cy7), CD4 (FITC), CD44 (APC-Cy7), NK1.1 (APC), CD3 (PE) and CD3 (PE-Texas Red), and analyzed them by multiparameter flow cytometry. NK1.1 and CD3 (cytoplasmic staining) expression were shown in (Fig. 3). Cytoplasmic CD3 expression was the highest in the DN_3_ cells (97%), then DN_2_ (83%), DN_4_ (74%) and DN_1_ cells (69%) (Fig. 3A) The flow cytometry data from more than three independent experiments performed in duplicate are shown (Fig. 3C). Cytoplasmic CD3 expression was highest in DP cells (99.5%), followed by CD8 (99.1%), CD4 (98.3%) and DN cells (81.2%) (Fig. 3B). The flow cytometry data from more than three independent experiments performed in duplicate are shown (Fig. 3D). These data suggest that cytoplasmic CD3 expressed in various stages of thymocytes.

### Maturational stages of NKT cells can be followed by differential expression of cytoplasmic and surface CD3 in thymocytes

**Figure 4:**
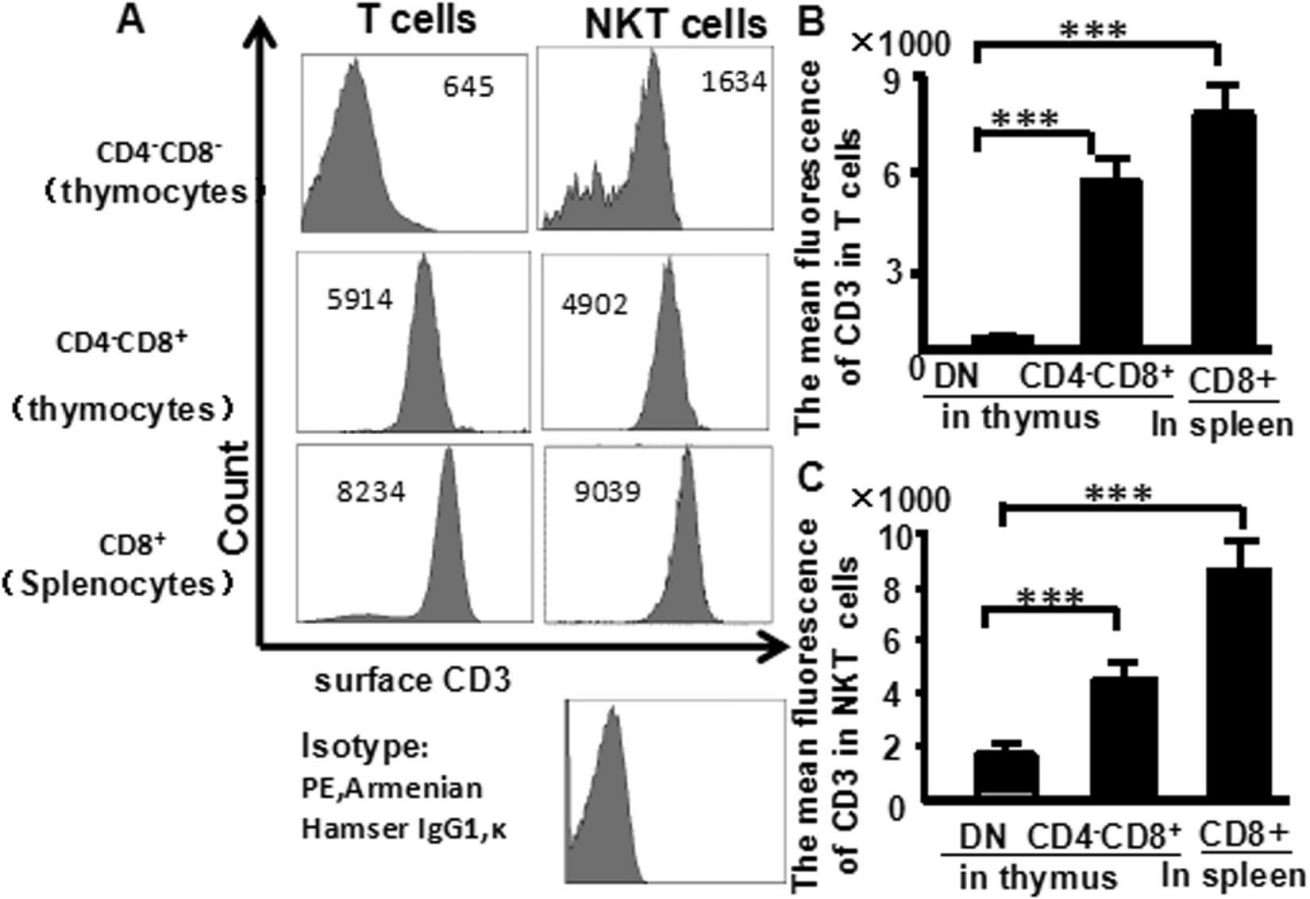
The fluorescence mean of surface CD3 expression in T and NKT thymocytes. Thymocytes from naive mice were stained with CD4 (FITC), CD8 (PerCP), CD25 (PE-Cy7), CD44 (APC-Cy7), NK1.1 (APC) and CD3 (PE), and analyzed by flow cytometry. (**A**) The fluorescence mean of surface CD3 in T cells of the thymus and spleen; (**B**) the fluorescence mean of surface CD3 in NKT cells of the thymus and spleen. Data were pooled from three independent experiments and shown in the plot. (**C**) The fluorescence mean of surface CD3 in T cells; (**D**) The fluorescence mean of surface CD3 in NKT cells. The data shows the percentage of total and live thymocytes in each cell subset, and are presented as the mean ± SD from three independent experiments. (***P < 0.001).

Cells from the murine thymus were stained with CD25 (PE-Cy7), CD4 (FITC), CD8 (PerCP), CD44 (APC-Cy7), NK1.1 (APC), CD3 (PE), and CD3 (PE-Texas Red) and then they were analyzed by flow cytometry. NK1.1 and surface CD3 expression were examined first (Fig. 4). The fluorescence intensity mean of surface CD3 in DN-T cells was significant lower (645) than in mature T cells (CD4-CD8+ cells in thymus: 8234 and CD8+ cells in spleen: 9039) (Fig. 4A). The fluorescence mean of surface CD3 in DN-NKT cells was significant lower (1634) than in mature NKT cells (CD4-CD8+ cells in thymus: 4902 and CD8+ cells in spleen: 9039) (Fig. 4B). The flow cytometry data from more than three independent experiments performed in duplicate is shown (Fig. 4C,D).

**Figure 5:**
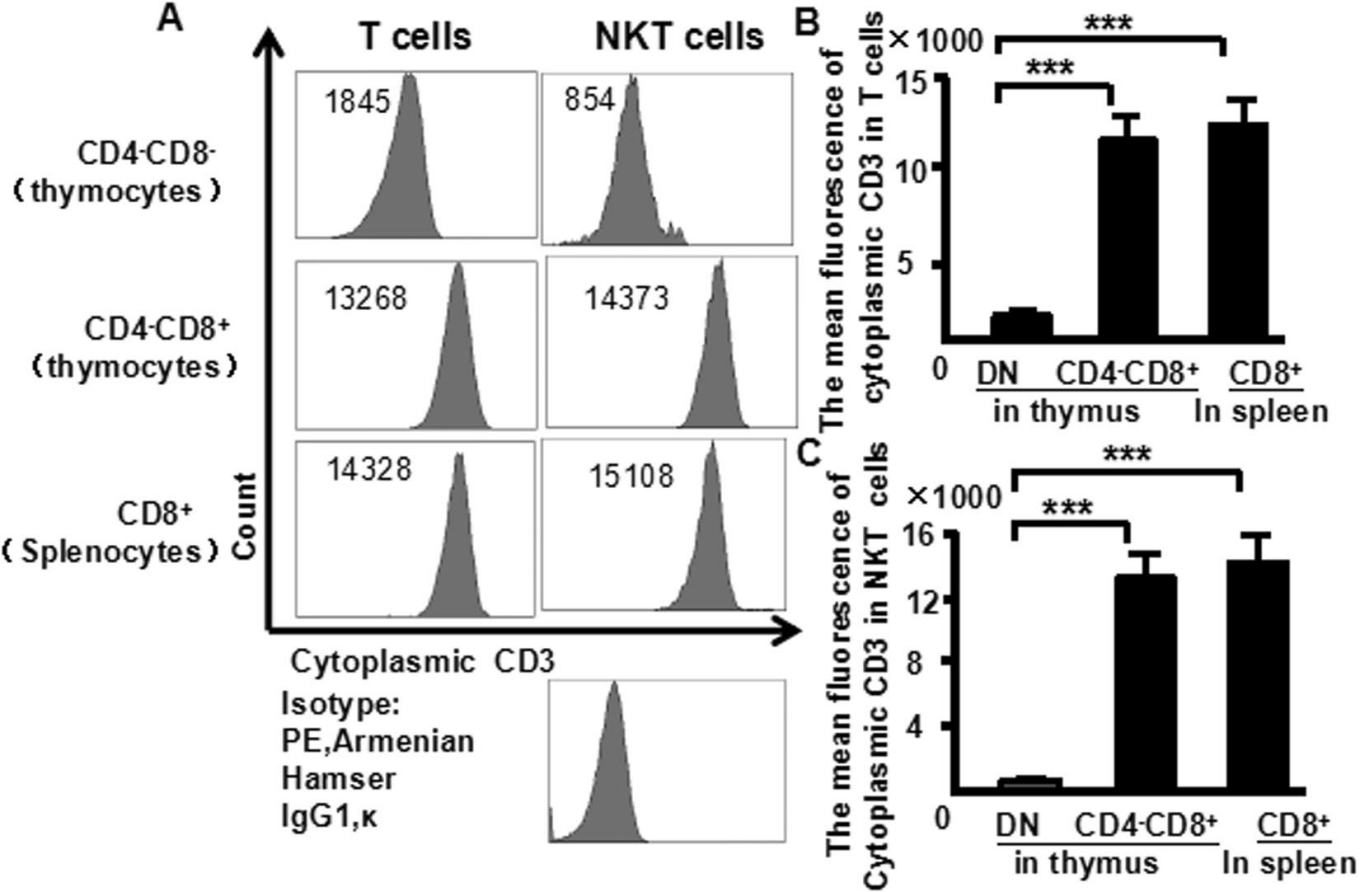
The fluorescence mean of cytoplasmic CD3 expression in T and NKT thymocytes. Thymocytes from naive mice were stained with CD4 (FITC), CD8 (PerCP), CD25 (PE-Cy7), CD44 (APC-Cy7), NK1.1 (APC) and CD3 (PE), and analyzed by flow cytometry. (**A**) The fluorescence mean of cytoplasmic CD3 in T cells of the thymus and spleen; (**B**) The fluorescence mean of cytoplasmic CD3 in NKT cells of the thymus and spleen. Data were pooled from three independent experiments and shown in the plot. (**C**) The fluorescence mean of cytoplasmic CD3 in T cells; (**D**) The fluorescence mean of cytoplasmic CD3 in NKT cells. The data shows the percentage of total and live thymocytes in each cell subset, and are presented as the mean ± SD from three independent experiments. (***P < 0.001).

Cytoplasmic CD3 expression was also examined for confirmation of our findings (Fig. 5). The fluorescence intensity mean of cytoplasmic CD3 in DN-T cells was significant lower (1845) than in mature T cells (CD4^−^CD8^+^ cells in thymus: 13,268 and CD8^+^ cells in spleen: 14,328) (Fig. 5A). The fluorescence intensity mean of cytoplasmic CD3 in DN-NKT cells was also significant lower (854) than in mature NKT cells (CD4-CD8+ cells in thymus: 14373 and CD8^+^ cells in spleen: 15108) (Fig. 5B). The flow cytometry data from more than three independent experiments is shown (Fig. 5C,D). These data suggest that the maturation states of T cells could be compared with the fluorescence intensity mean of cytoplasmic and surface CD3. The NKT cells in DN thymocytes showed the least mean fluorescence intensity of cytoplasmic and surface CD3 within thymocytes.

**Figure 6:**
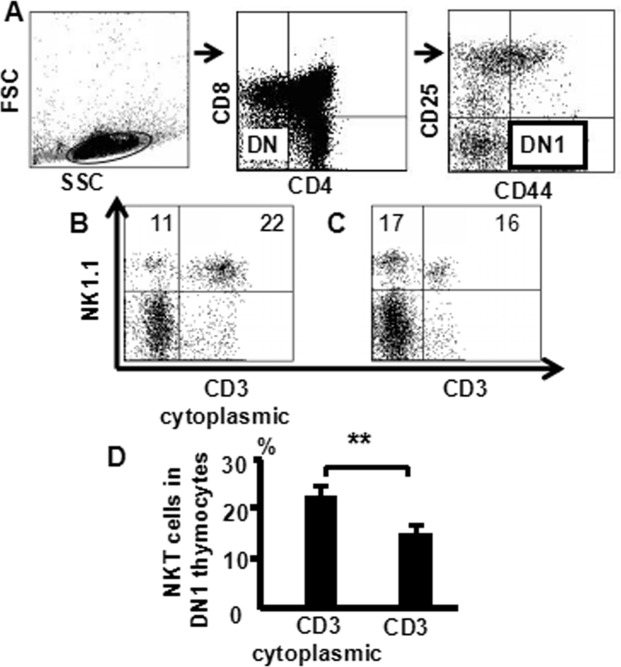
The number of cytoplasmic CD3-NKT and surface CD3-NKT cells in DN-thymocytes. Thymocytes from naive mice were stained with CD4 (FITC), CD8 (PerCP), CD25 (PE-Cy7), CD44 (APC-Cy7), NK1.1 (APC) and CD3 (PE, intracellular and extracellular staining), and analyzed by flow cytometry. (**A**) DN_1_ cells were gated from DN thymocytes; (**B**) Cytoplasmic CD3-NKT in DN_1_-thymocytes; (**C**) Surface CD3-NKT in DN_1_-thymocytes. (**D**) The pooled data from three independent experiments. The data shows the percentage of total and live thymocytes in each cell subset, and are presented as the mean ± SD from three independent experiments. (**P < 0.01).

Next, to confirm NKT cells in DN thymocytes are immature cells, cells from the murine thymus and stained with CD8 (PerCP), CD25 (PE-Cy7), CD4 (FITC), CD44 (APC-Cy7), NK1.1 (APC), CD3 (PE) and CD3 (PE-Texas Red), and analyzed them by multiparameter flow cytometry. NK1.1 and CD3 (cytoplasmic staining and surface staining) expression were shown in (Fig. 6). We first gated live thymocytes by size (FSC) and granularity (SSC), followed by dividing the live cells into four subpopulations based on CD4 and CD8 expression, and CD4^−^CD8^−^ cells (DN) were gated. Since the cells had also been stained with antibodies against CD44 and CD25, CD44^+^CD25^−^(DN_1_), cells were gated (Fig. 6A); cytoplasmic CD3- NKT cells 22% in DN_1_ thymocytes (Fig. 6B); surface CD3-NKT cells only 16% (Fig. 6C). Pooled data from three independent experiments is shown (Fig. 6D). These data suggest that there are not only mature (surface CD3^+^) NKT cells but also immature (surface CD3^−^ cytoplasmic CD3^+^) NKT cells in DN cells.

### TCR-β and CD3 expression in DN_1_-NKT thymocytes

**Figure 7:**
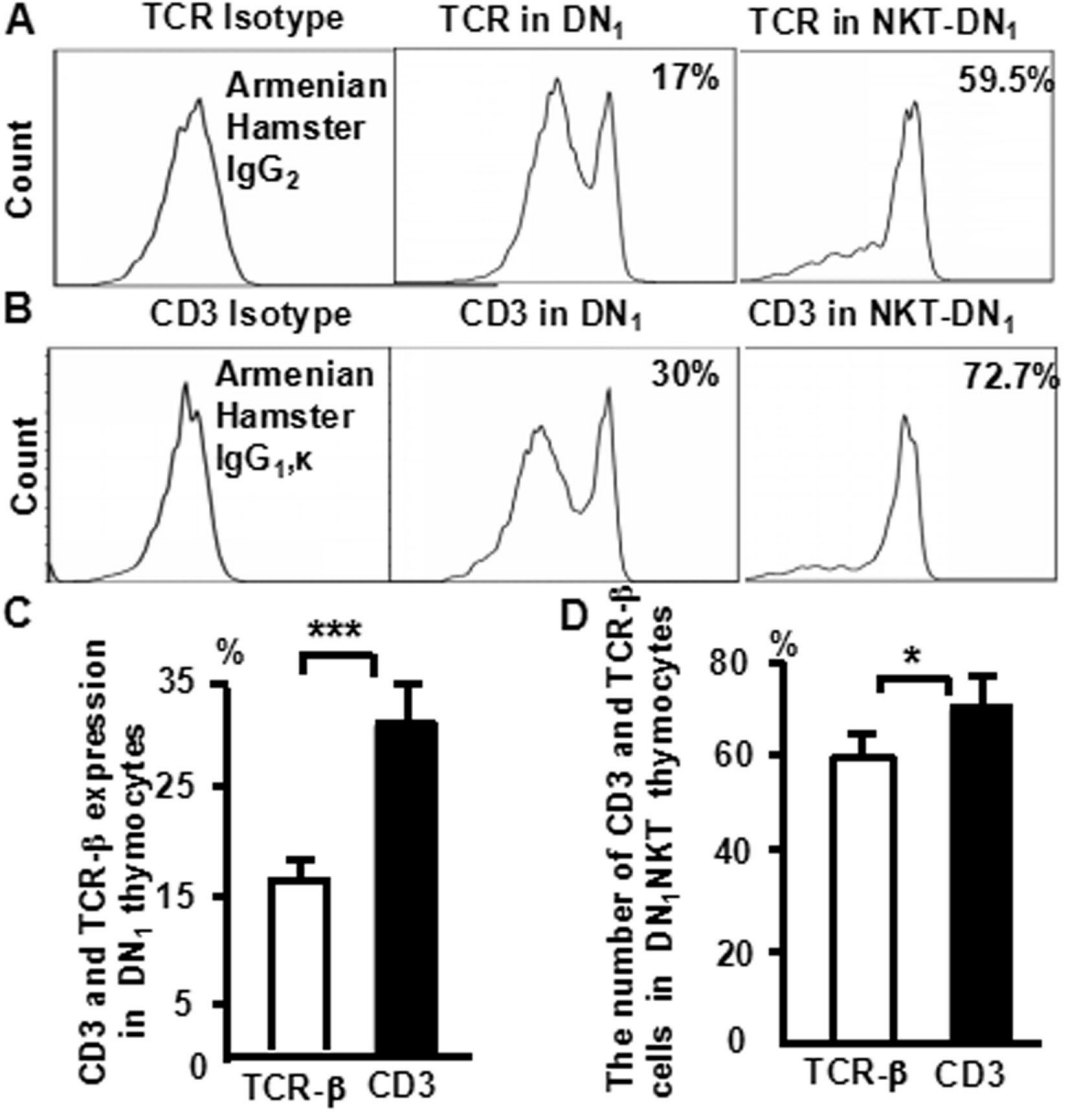
TCR-β and CD3 expression in DN_1_-NKT thymocytes. Thymocytes were collected from naïve mice, stained with antibodies against CD4, CD8, CD44, CD25, CD3, NK1.1 and TCR-β, and then analyzed by flow cytometry. (**A**) TCR-β and CD3 expression in DN_1_ thymocytes; (**B**) The number of TCR-β-NK1.1 and CD3-NK1.1 cells in DN_1_ thymocytes; Data were pooled from three independent experiments and shown in the plot (**C**,**D**). The data shows the percentage of total and live thymocytes in each cell subset, and are presented as the mean ± SD from three independent experiments. (***P < 0.001,*P < 0.05).

**Figure 8:**
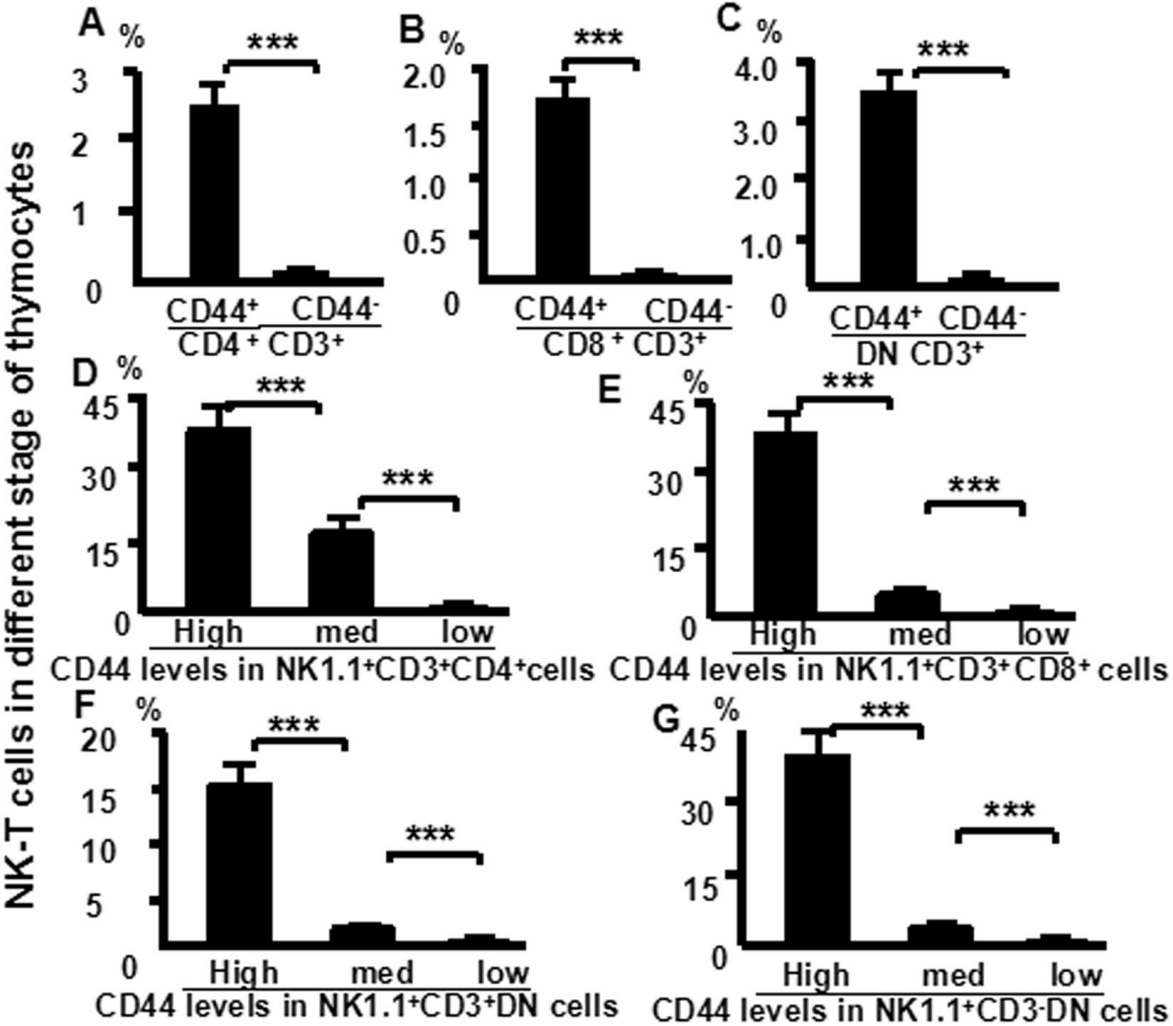
NK1.1 expression positively correlates with CD44 expression in NKT thymocytes. Cells were stained with CD4 (FITC), CD8 (PerCP), CD44 (APC-CY7), CD25 (PE-Cy7), NK1.1 (APC) and CD3 (PE) and then analyzed by flow cytometry. (**A**) the number of NKT cells in CD44^+^ and CD44^−^ of CD4 cells; (**B**) in CD44^+^ and CD44^−^ of CD8 cells; (**C**) in CD44^+^ and CD44^−^ of DN cells. (**D**) CD44 levels in NK1.1^+^CD3^+^ CD4 cells; (**E**) CD44 levels in NK1.1^+^CD3^+^ CD8 cells; (**F**) CD44 levels in NK1.1^+^CD3^+^ DN cells; (**G**) CD44 levels in NK1.1^+^CD3^−^ DN cells. The data shows the percentage of total and live thymocytes in each cell subset, and are presented as the mean ± SD from three independent experiments. (***P < 0.001).

Since a productive TCR-β gene rearrangement is a critical event in thymocyte development and proliferation^34,35^, we then examined TCR-β and CD3 expression in DN_1_ thymocytes, cells from the murine thymus were stained with CD8 (PerCP), CD25 (PE-Cy7), CD44 (APC-Cy7), CD4 (FITC), NK1.1(APC),TCR**-**β (PE) and CD3 (PE-Texas Red), and analyzed them by multiparameter flow cytometry. TCR-β and CD3 expressions were shown in (Fig. 7). CD3 expression (30%) was much higher than TCR-β (17%) in DN_1_ thymocytes (Fig. 7A). The flow cytometry data from more than three independent experiments performed in duplicate is shown (Fig. 7C). The number of CD3-NK1.1 cells (72.7%) was higher than TCR-β-NK1.1 cells (59.5%) in DN_1_-NKT thymocytes (Fig. 7B). The flow cytometry data from more than three independent experiments performed in duplicate is shown (Fig. 7D). These data suggest that there are not only mature T cells but also immature T cells in surface CD3 positive DN_1_ thymocytes that do not express TCR-β, some of them are immature (surface CD3^+^ TCR-β^−^) NKT cells. NK1.1 expression is correlated with CD44 expression in NKT cells. To determine whether NK1.1 expression correlated with CD44 expression, cells from the murine thymus were stained with various antibodies and analyzed by flow cytometry. There were more NKT cells in CD44+ CD4+, CD44+ CD8+, and CD44+ DN cells (2.5%, 1.75%, and 3.6%) than in CD44−CD4+, CD44-CD8+, and CD44− DN cells (0%) (Fig. 8A–C). We also observed a correlation between CD44 and NK1.1 expression in NKT cells, wherein the level of NK1.1 was highest in CD4+ CD44high cells (38%), then CD4+ CD44med (17%) and CD4+ CD44low cells (0%) (Fig. 8D). The level of NK1.1 was also high in CD8+ CD44high cells (40%), followed by CD8+ CD44med (4.2%) and CD8+ CD44low (0%) cells (Fig. 8E). Similarly, the level of NK1.1 was high in DN-CD3+ CD44 high cells (15.8%), followed by DN-CD3 +CD44 med (2%) and DN-CD3+ CD44 low cells (0%) (Fig. 8F). The level of NK1.1 was high in DN-CD3-CD44high cells (40.6%), followed by DN-CD3-CD44 med (3.5%) and DN-CD3-CD44low (0%) cells (Fig. 8G). Thus, we found a positive correlation between NK1.1 and CD44 expression among NKT cells, suggesting that CD44 may be an additional marker for NKT cells.

### IL-7Rα expression correlated with CD44 expression in NKT cells

**Figure 9:**
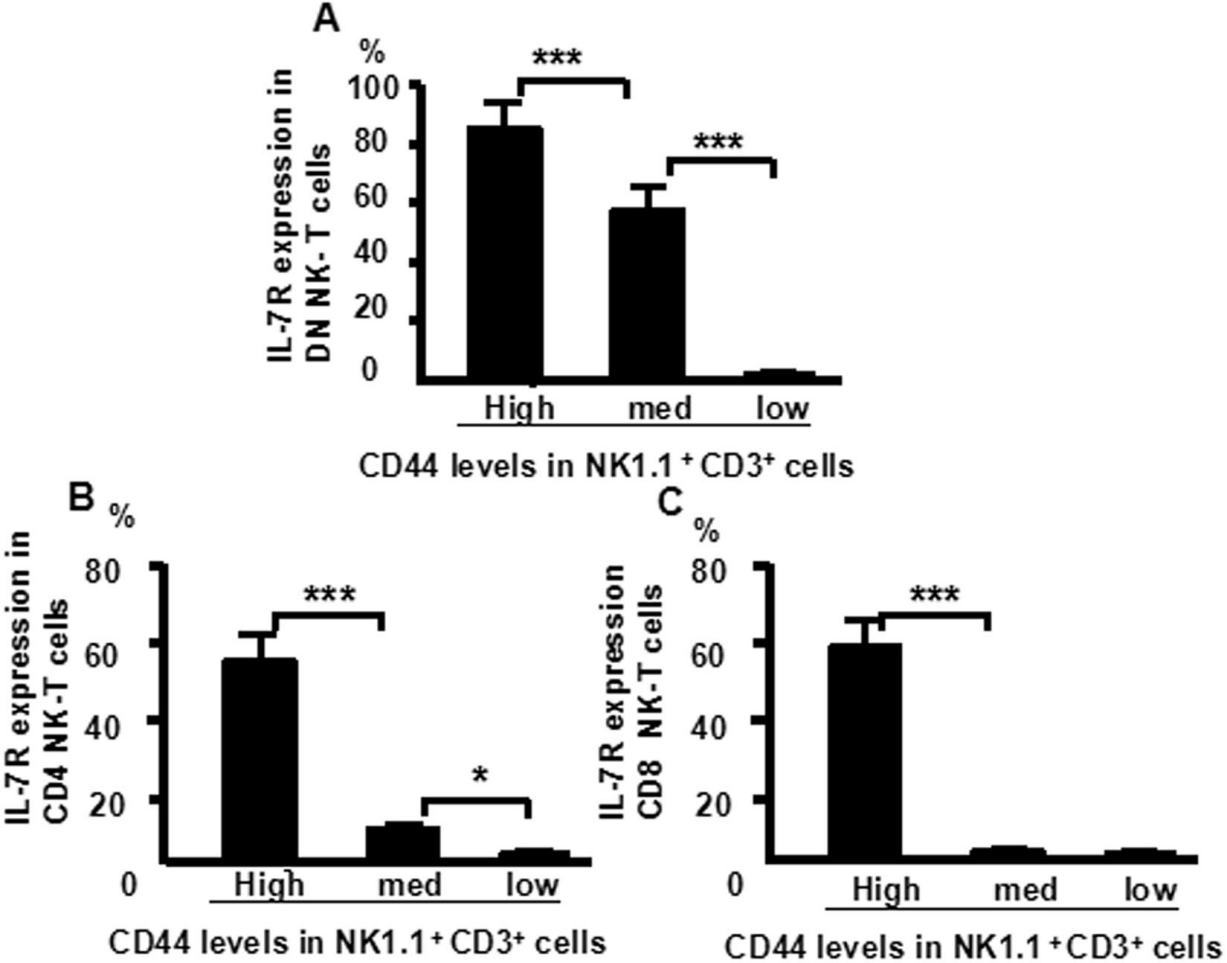
IL-7R expression positively correlates with CD44 expression in CD3^+^ thymocytes. Cells were stained with CD4 (FITC), CD8 (PerCP), CD44 (APC-Cy7), CD25 (PE-Cy7), NK1.1 (APC), L-7Rα (PE) and CD3 (PE-Texas-Red), and the level of IL-7Rα expression analyzed by flow cytometry. (**A**) CD44 levels in DN-NK1.1^+^CD3^+^ cells; (**B**) CD44 levels in CD4^+^NK1.1^+^CD3^+^ cells; (**C**) CD44 levels in CD8^+^NK1.1^+^CD3^+^cells. The data shows the percentage of total and live thymocytes in each cell subset, and are presented as the mean ± SD from three independent experiments (***P < 0.001, *P < 0.05).

Cells from the murine thymus were stained with CD8 (PerCP), CD25 (PE-Cy7), CD4 (FITC), CD44 (APC-Cy7), NK1.1 (APC) and IL-7Rα (PE), CD3 (PE- Texas-red) and analyzed by flow cytometry. IL-7Rα expression was highest in DN^−^NK1.1^+^CD3^+^CD44^high^ cells (86%), followed by CD44^med^ (57%) and CD44^low^ (0%) cells (Fig. 9A). Similarly, IL-7Rα expression was high in CD4^+^NK1.1^+^CD3^+^ CD44^high^ cells (56%), followed by CD44^med^ (11.3%) and CD44^low^ (0.1%) cells (Fig. 9B), and IL-7Rα expression was also high in CD8^+^NK1.1^+^CD3^+^CD44^high^ cells (59%), followed by CD44^med^ (1.6%) and CD44^low^ (0.1%) cells (Fig. 9C). These results suggest that IL-7Rα expression was correlated with CD44 expression in NKT cells of thymocytes.

### IL-7Rα expression in NKT and T cells of thymus and spleen

**Figure 10:**
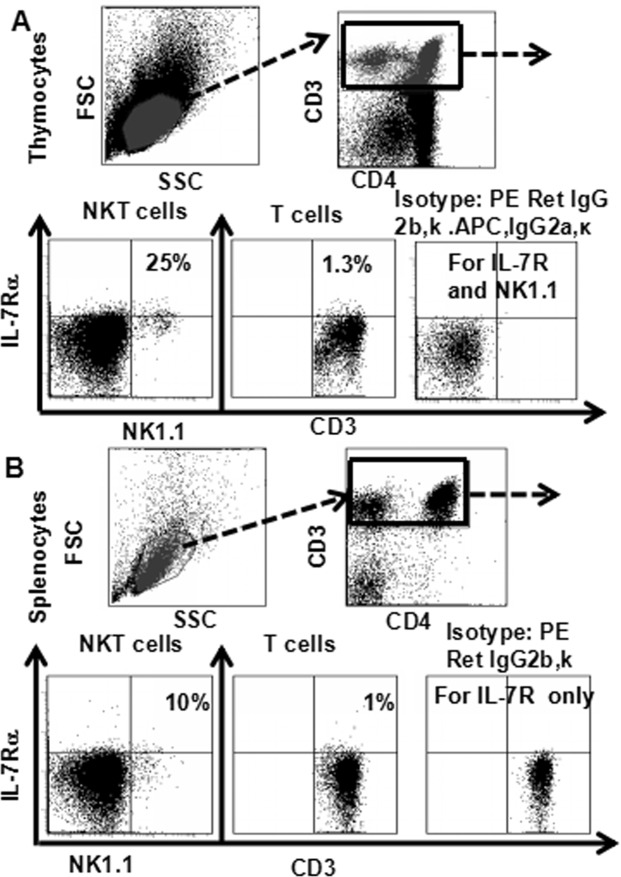
IL-7Rα expression in NKT and T cells in thymus and spleen. Cells were incubated in medium containing IL-7 or LPS for 24 hours, and stained with CD4 (FITC), CD8 (PerCP), NK1.1(APC), IL-7Rα (PE) and CD3 (PE-Texas-Red), and the level of IL-7Rα expression was analyzed by flow cytometry. (**A**) IL-7Rα in NKT and T cells in thymus; (**B**) IL-7Rα in NKT and T cells in spleen. The data shows the percentage of total and live thymocytes in each cell subset, and are presented as the mean ± SD from three independent experiments.

Cells from murine thymuses and spleens were stained with CD8 (PerCP), CD25 (PE-Cy7), CD4 (FITC), CD44 (APC-Cy7), NK1.1 (APC) and IL-7Rα (PE), CD3 (PE- Texas-red) and analyzed them by flow cytometry (Fig. 10A). NKT cells expressed higher levels of IL-7Rα (25%, 10%) than T cells (1.3%, 1%) in thymocytes and splenocytes (Fig. 10B). These results suggested that NKT cells are more sensitive to IL-7/IL-7Rα signaling regulation than T cells.

## Discussion

In this study, we used an advanced flow cytometry method to explore NKT cell development in the thymus. We examined changes in the expression of CD25, CD44, CD3, and NK1.1 in thymocytes. The use of specific mAbs allowed us to analyze NKT cells in the thymus. The level of NK1.1 expression was highest in CD4^−^CD8^−^ (DN) cells, after that CD4^+^CD8^−^ and CD4^+^CD8^+^ cells (Fig. 1). The level of NK1.1expression was highest in DN_1_ cells, after that DN_2_, while DN_3_ and DN_4_ cells did not express NK1.1 (Fig. 2A). Similarly, the number of NKT cells was highest in DN_1_cells, and could not be identified in DN_2_, DN_3_, and DN_4_ cells (Fig. 2B). Cytoplasmic CD3 was expressed in various stages of DN thymocytes and in various stages of thymocytes (Fig. 3). This result suggests that cytoplasmic CD3 could be used as a marker for early stage of T cells. From the above research, we found that mature T cells expressed both surface CD3 and cytoplasmic CD3, whereas immature T cells only expressed cytoplasmic CD3. The fluorescence mean intensities of CD3 in T and NKT cells within the DN thymocytes were significantly lower than mature T and NKT cells (Figs 4 and 5), suggests that the maturation states of T cells could be compared with the fluorescence mean of cytoplasmic CD3 and surface CD3.NKT cells in DN thymocytes showed the least fluorescence intensity mean of cytoplasmic and surface CD3 within thymocytes. T cells expressing CD3 did not show that it must be mature cells, such as CD3^+^DN-T cells and CD3^+^DN-NKT cells, which express weak strength of the fluorescence mean CD3 are immature cells. The number of NKT cells in DN-cytoplasmic CD3 was higher than in DN- surface CD3 cells (Fig. 6), this result suggest at least part of NKT cells in DN thymocytes are immature cells. CD3 expression was higher than TCR-β in DN_1_ thymocytes. The number of CD3-NK1.1 cells was higher than TCR-β-NK1.1 cells in DN_1_ thymocytes (Fig. 7). These results suggest that there are not only mature T cells but also immature T cells in surface CD3 positive DN_1_ thymocytes, some of them do not express TCR-β and are immature NKT cells. NKT cells development may from DN thymocytes. NK1.1 expression and the number of NKT cells were correlated with CD44 expression in the murine thymus (Fig. 8). The surface IL-7Rα expression correlates with NK1.1, and CD44 expression (Fig. 9). Additionally, IL-7Rα was expressed at much higher levels in NKT cells than in T cells (Fig. 10). The discovery that there was more IL-7Ra expression in NKT cells than in conventional T cells is important with potential clinical implication. This would imply that IL-7Ra deficient patients would suffer from a more profound NKT deficiency than originally thought. We recently studied T_reg_ cells and examined Foxp3 expression in Thymus by flow cytometry and found T_reg_ cell development does not accord with the theory of DN T cell development (DN_1_, DN_2_, DN_3_, DN_4_), nor the theory of T-cell development (DN, DP, SP). In the present study, we found that NKT cells like T_reg_ cells develop in the thymus. NKT cell development may involve transition from DN-T progenitor cells directly to SP cells. Surprisingly, both NKT cells and T_regs_ are the phenotype of T cell; yet do not share the T cell development pathway. Although NKT cells are present in the thymus, the developmental origin of these cells are controversial. Previous studies showed that NKT cell development was probably from CD8 cells^36^, DP cells^12,37^, CD4 cells^38,39^, and BM cells^40^. However in the present study, we found that NKT cells within DN thymocytes, much like DN T cells, expressed lower levels of fluorescence mean of CD3. DN_2_ and DN_3_ did not express surface CD3, but expressed higher level of cytoplasmic CD3. There is a gradual expression of CD3 from weak to strong and from intracellular to surface in T cell development, especially in the early stage, although some cells weakly express CD3 but are immature cells, such as in CD3 (30%), TCRβ (17%) in DN_1_ thymocytes (Fig. 7C). If TCR-β is the marker for mature T cells, then half of CD3 T cells could be classified as immature in DN_1_ thymocytes; therefore, it is unlikely that all CD3-expressing cells are mature cells. There are not only mature T cells but also immature among cells in surface CD3 positive DN_1_ thymocytes. Some of them do not express TCR-β and are immature surface CD3^+^TCR-β^−^NKT cells (Fig. 7D). Furthermore, the number of cytoplasmic CD3-NKT cells was significantly higher than the surface of CD3-NKT cells in DN_1_ thymocytes, which also suggest that NKT cells within DN thymocytes are immature cells, at least some of them are immature (cytoplasmic CD3^+^ surface CD3^−^) NKT cells. Although previous studies have indicated that DN-NKT cells are mature cells, this conjecture is only derived from indirect evidence, strong direct evidence is still needed to confirm this observation. If you want to prove that NKT cells derived from mature cells, you must to prove that no immature cells in the DN thymocytes. Our study has proved very clear that the development of NKT can be derived from the immature cytoplasmic CD3^+^ surface CD3^−^ and surface CD3^+^TCR-β^−^DN_1_ thymocytes. In this study, we found a higher population of NKT cells are immature cells in the DN_1_ stage. NKT cells may develop from DN progenitor, involved the cytoplasmic CD3^+^ DN_1_, DN_2_ stage, yet without the involvement of the DN_3_, DN_4_, and CD4^+^CD8^+^(DP) stages. We propose that NKT cells in mice may development originate from cytoplasmic CD3 positive, CD44 and IL-7Rα expressing DN thymocytes.

## Supplementary information

NKT Cells in Mice Originate from Cytoplasmic CD3-Positive, CD4-CD8- Double-Negative Thymocytes that Express CD44 and IL-7Rα

## References

[1] Bendelac A, Savage PB, Teyton L (2007). The biology of NKT cells. Annu Rev Immunol.

[2] Taniguchi M, Seino K, Nakayama T (2003). The NKT cell system: bridging innate and acquired immunity. Nat Immunol.

[3] Wu L, Van Kaer L (2009). Natural killer T cells and autoimmune disease. Curr Mol Med.

[4] Godfrey DI, Hammond KJ, Poulton LD, Smyth MJ, Baxter AG (2000). NKT cells: facts, functions and fallacies. Immunol Today.

[5] Yoshimoto T, Paul WE (1994). CD4pos, NK1.1pos T cells promptly produce interleukin 4 in response to *in vivo* challenge with anti-CD3. J Exp Med.

[6] Bendelac A, Rivera MN, Park SH, Roark JH (1997). Mouse CD1-specific NK1 T cells: development, specificity, and function. Annu Rev Immunol.

[7] Cui J (1997). Requirement for Valpha14 NKT cells in IL-12-mediated rejection of tumors. Science.

[8] Balciunaite G, Ceredig R, Rolink AG (2005). The earliest subpopulation of mouse thymocytes contains potent T, significant macrophage, and natural killer cell but no B-lymphocyte potential. Blood.

[9] Porritt HE (2004). Heterogeneity among DN1 prothymocytes reveals multiple progenitors with different capacities to generate T cell and non-T cell lineages. Immunity.

[10] Carlyle JR, Zuniga-Pflucker JC (1998). Lineage commitment and differentiation of T and natural killer lymphocytes in the fetal mouse. Immunol Rev.

[11] Spits H (1998). Early stages in the development of human T, natural killer and thymic dendritic cells. Immunol Rev.

[12] Hager E, Hawwari A, Matsuda JL, Krangel MS, Gapin L (2007). Multiple constraints at the level of TCRalpha rearrangement impact Valpha14i NKT cell development. J Immunol.

[13] Sato H (1999). Induction of differentiation of pre-NKT cells to mature Valpha14 NKT cells by granulocyte/macrophage colony-stimulating factor. Proc Natl Acad Sci USA.

[14] Alarcon B, Gil D, Delgado P, Schamel WW (2003). Initiation of TCR signaling: regulation within CD3 dimers. Immunol Rev.

[15] Letourneur F, Klausner RD (1992). A novel di-leucine motif and a tyrosine-based motif independently mediate lysosomal targeting and endocytosis of CD3 chains. Cell.

[16] Marks MS, Woodruff L, Ohno H, Bonifacino JS (1996). Protein targeting by tyrosine- and di-leucine-based signals: evidence for distinct saturable components. J Cell Biol.

[17] Wilson. A, MacDonald HR (1995). Expression of genes encoding the pre-TCR and CD3 complex during thymus development. Int Immunol.

[18] Brodeur JF, Li S, Da Silva Martins M, Larose L, Dave VP (2009). Critical and multiple roles for the CD3epsilon intracytoplasmic tail in double negative to double positive thymocyte differentiation. J Immunol.

[19] Levelt CN, Carsetti R, Eichmann K (1993). Regulation of thymocyte development through CD3. II. Expression of T cell receptor beta CD3 epsilon and maturation to the CD4+ 8+ stage are highly correlated in individual thymocytes. J Exp Med.

[20] Wilson A, Capone M, MacDonald HR (1999). Unexpectedly late expression of intracellular CD3epsilon and TCR gammadelta proteins during adult thymus development. Int Immunol.

[21] Kane LP, Lin J, Weiss A (2000). Signal transduction by the TCR for antigen. Curr Opin Immunol.

[22] Kuhns MS, Davis MM (2012). TCR Signaling Emerges from the Sum of Many Parts. Front Immunol.

[23] Jacobs SR, Michalek RD, Rathmell JC (2010). IL-7 is essential for homeostatic control of T cell metabolism *in vivo*. J Immunol.

[24] Boesteanu A (1997). Distinct roles for signals relayed through the common cytokine receptor gamma chain and interleukin 7 receptor alpha chain in natural T cell development. J Exp Med.

[25] Matsuda JL (2002). Homeostasis of V alpha 14i NKT cells. Nat Immunol.

[26] Nandi A, Estess P, Siegelman MH (2000). Hyaluronan anchoring and regulation on the surface of vascular endothelial cells is mediated through the functionally active form of CD44. J Biol Chem.

[27] Khaldoyanidi S, Denzel A, Zoller M (1996). Requirement for CD44 in proliferation and homing of hematopoietic precursor cells. J Leukoc Biol.

[28] Poynter ME, Mu HH, Chen XP, Daynes RA (1997). Activation of NK1.1+ T cells *in vitro* and their possible role in age-associated changes in inducible IL-4 production. Cell Immunol.

[29] Liu T, Soong L, Liu G, Konig R, Chopra AK (2009). CD44 expression positively correlates with Foxp3 expression and suppressive function of CD4+ Treg cells. Biol Direct.

[30] Hayday AC, Pennington DJ (2007). Key factors in the organized chaos of early T cell development. Nat Immunol.

[31] Takahama Y (2006). Journey through the thymus: stromal guides for T-cell development and selection. Nat Rev Immunol.

[32] Gigliotti D, Nihlmark EL, Wigzell H, Hansson M (1993). Murine thymocytes with ability to inhibit IL-2 production. II. Characterization of a subpopulation with regulatory function in the thymus. Cell Immunol.

[33] Liu G (2014). Direct Detection of FoxP3 Expression in Thymic Double-Negative CD4(−)CD8(−) Cells by Flow Cytometry. Sci Rep.

[34] Germain RN (2002). T-cell development and the CD4-CD8 lineage decision. Nat Rev Immunol.

[35] Bosselut R (2004). CD4/CD8-lineage differentiation in the thymus: from nuclear effectors to membrane signals. Nat Rev Immunol.

[36] Assarsson E (2000). CD8+ T cells rapidly acquire NK1.1 and NK cell-associated molecules upon stimulation *in vitro* and *in vivo*. J Immunol.

[37] Coles MC, Raulet DH (2000). NK1.1+ T cells in the liver arise in the thymus and are selected by interactions with class I molecules on CD4+ CD8+ cells. J Immunol.

[38] Pellicci DG (2002). A natural killer T (NKT) cell developmental pathway iInvolving a thymus-dependent NK1.1(−)CD4(+) CD1d-dependent precursor stage. J Exp Med.

[39] Coles MC, Raulet DH (1994). Class I dependence of the development of CD4+ CD8− NK1.1+ thymocytes. J Exp Med.

[40] Masuda K (2005). Thymic anlage is colonized by progenitors restricted to T, NK, and dendritic cell lineages. J Immunol.

