# Supplementary material for: NKT Cells in Mice Originate from Cytoplasmic CD3-Positive, CD4−CD8− Double-Negative Thymocytes that Express CD44 and IL-7Rα
Source: Sci Rep. 2019 Feb 12;9:1874. doi: 10.1038/s41598-018-37811-0 (PMC6372634; doi:10.1038/s41598-018-37811-0)
Supplement: Supplementary file 1 — NKT Cells in Mice Originate from Cytoplasmic CD3-Positive, CD4-CD8- Double-Negative Thymocytes that Express CD44 and IL-7Rα [file 41598_2018_37811_MOESM1_ESM.pdf]

NKT Cells in Mice Originate from Cytoplasmic CD3-Positive, CD4^{-}CD8^{-} Double-Negative Thymocytes that Express CD44 and IL-7Rα

Zhansheng Hu, Wen Gu, Yang Wei , Gang Liu, Shengli Wu· , Tie Liu

Supplementary Information

1. Supplementary Figures;
2. Detection of TCR-β in DN cells by improved flow cytometry;
3. Detection of TCR-βon different stages of DN cells by improved flow cytometry.

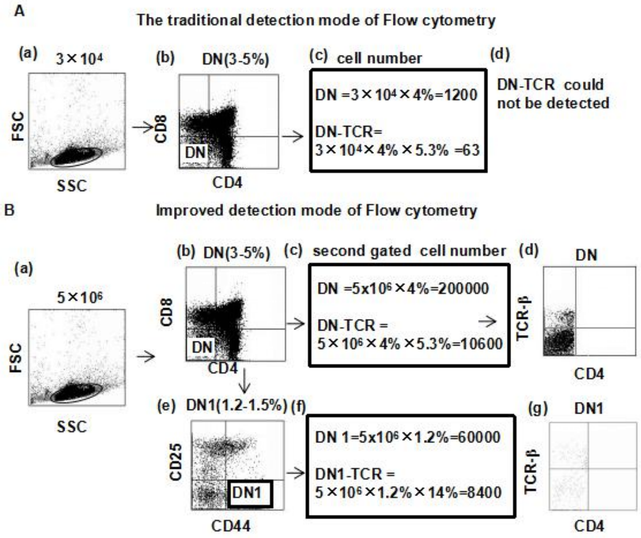

Figure S1. Detection of TCR-β on different stages of DN cells by improved flow cytometry. Thymocytes were collected from naïve mice, stained with antibodies against CD4, CD8, CD44, CD25 and TCR-β , and then analyzed by flow cytometry. (A) TCR- β expression by traditional flow cytometry; (B) TCR- β expression by improved flow cytometry.

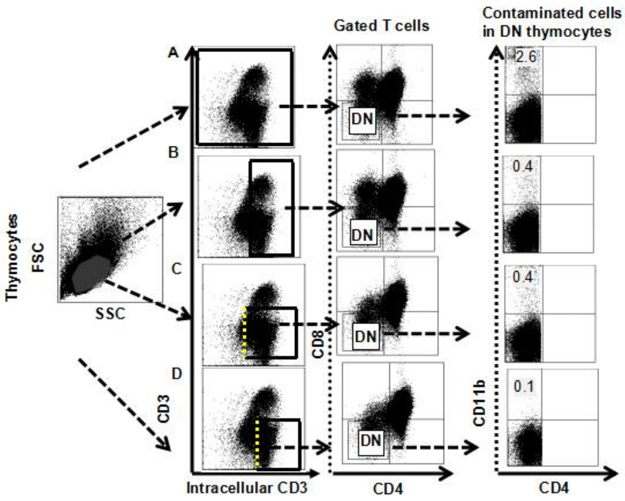

Figure S2. Removal of contaminated cells by the intracellular CD3-gating. thymocytes from naive mice were stained with CD4 (FITC), CD8 (PerCP), CD25 (PE-Cy7), CD44 (APC-Cy7), NK1.1 (APC), CD3 (PE), CD3 (PE-Texas red) and analyzed them by flow cytometry; (A) whole cells gated; (B) intracellular CD3 and CD3 cells gated; (C) intracellular CD3 cells gated; (D) intracellular CD3^{med} and intracellular CD3^{high} cells gated.

Detection of TCR-β in DN cells by improved flow cytometry. TCR regulate T-cell proliferation and differentiation in the thymus and the periphery. While flow cytometry is one of the most important research methods for examining T cell development, traditional flow cytometry cannot accurately predict percentages for cells that are expressed below a certain percentage or a certain amount. CD4$^-$CD8$^-$ DN

cells comprise only 3–5% of the total thymocytes within the thymus [32]. Furthermore, the expression of many genes, such as the *TCR-β* gene is very low in DN thymocytes (5.3%); therefore, accurate detection of protein molecules in DN cells by flow cytometry is challenging. As shown in **Figure S1A**, the traditional methodology of flow cytometry with 3×10^4 cells only **Figure S1A**(**a**) yields approximately 1200 DN cells ($3\times10^4\times4\%$), with only 63 ($3\times10^4\times4\%\times5.3\%$) DN-TCR-β cells **Figure S1A** (**b**). DN-TCR could not be detected **Figure S1A** (**c**). However, we improved the detection capabilities resulting in increased cell numbers collected for each sample (from 10^4 to 5×10^6) **Supplementary Figure S1B** (**a**). This brought the number of DN cells up to 200000 ($5\times10^6\times4\%$) =**Figure S1B** (**b**). Consequently, the number of DN-TCR-β cells could be 10600 ($5\times10^6\times4\%\times5.3\%$) **Figure S1B** (**c, d**). Because DN_1 cells comprise approximately 1.2% of thymocytes and approximately 14% of DN_1 cells are TCR-β-positive **Figure S1B** (**e**), we were able to detect $5\times10^6\times1.2\%\times14\%=8400$ DN_1-TCR-β cells by this method **Figure S1B** (**f, g**). Therefore, using this improved the flow cytometry detection method (5×10^6 thymocytes were collected for each sample). Moreover, lower expression protein molecules in each subpopulation of DN cells could be detected to reveal previously uncharacterized data on subsets of DN cells. In order to improve the detection efficiency, we ensured the following features: (i) we used FACSAria III to ensure the stability of acquisition; (ii) each collection was performed for less than 1000 events (60-80min/each sample); and (iii), each color channel in the instrument (multiple-color flow cytometry) was accurately calibrated by maintenance engineers.

Removal of contaminated cells by intracellular CD3 gating. Traditionally, contaminated cells (non–T-cell lineages) must be removed by specific blocking antibodies before detection of DN cells. We found intracellular CD3 was expressed in the majority of DN thymocytes, and removed contaminated cells by the intracellular CD3 gated (a detection software technology of flow cytometry) and then analyzed protein molecules in DN thymocytes. Four methods can be used to detect the DN thymocytes and remove contaminated cells (such as CD11b): (i) gating all the thymocytes and then remove the contaminated cells by calculation of data (2.6%) to correct experimental results **(Figure S2A)**; (ii) removal of contaminated cells (0.4%) by the intracellular CD3 and CD3 gating **(Figure S2B)**; (iii) removal of contaminated cells (0.4%) by the intracellular CD3 gating only**(Figure S2C)**; and (iv) some intracellular CD3 cells expressed contaminated cell markers, such as the level of CD11b was higher in DN intracellular $CD3^{low}$ cells, therefore we gated intracellular $CD3^{med}$ and $CD44^{high}$ cells, and identified the contaminated cells (0.1%) (**Figure S2D)**.
